# Co-regulation of photosynthetic capacity by nitrogen, phosphorus and magnesium in a subtropical Karst forest in China

**DOI:** 10.1038/s41598-018-25839-1

**Published:** 2018-05-09

**Authors:** Jing Wang, Xuefa Wen, Xinyu Zhang, Shenggong Li, Da-Yong Zhang

**Affiliations:** 1 Key Laboratory of Ecosystem Network Observation and Modeling, Institute of Geographic Sciences and Natural Resources Research, Chinese Academy of Sciences, Beijing, 100101 China; 2 College of Resources and Environment, University of Chinese Academy of Sciences, Beijing, 100190 China; 3 School of Life Sciences, Beijing Normal University, Beijing, 100875 China

## Abstract

Leaf photosynthetic capacity is mainly constrained by nitrogen (N) and phosphorus (P). Little attention has been given to the photosynthetic capacity of mature forests with high calcium (Ca) and magnesium (Mg) in the Karst critical zone. We measured light-saturated net photosynthesis (*A*_sat_), photosynthetic capacity (maximum carboxylation rate [*V*_cmax_], and maximum electron transport rate [*J*_max_]) as well as leaf nutrient contents (N, P, Ca, Mg, potassium [K], and sodium [Na]), leaf mass per area (*LMA*), and leaf thickness (*LT*) in 63 dominant plants in a mature subtropical forest in the Karst critical zone in southwestern China. Compared with global data, plants showed higher *A*_sat_ for a given level of P. *V*_cmax_ and *J*_max_ were mainly co-regulated by N, P, Mg, and *LT*. The ratios of *V*_cmax_ to N or P, and *J*_max_ to N or P were significantly positively related to Mg. We speculate that the photosynthetic capacity of Karst plants can be modified by Mg because Mg can enhance photosynthetic N and P use efficiency.

## Introduction

The highly sensitive Karst Critical Zones (CZs) account for about 12% of the global terrestrial land area^1^, with more than 54 × 10^4^ km^2^ distributed in southwestern China^2^. The Critical Zone (CZ) is defined by the US National Research^3^ as “*a heterogeneous, near surface environment in which complex interactions involving rock, soil, water, air and living organisms regulate the natural habitat and determine availability of life sustaining resources*.” Compared with other CZs, Karst CZs were developed on limestone, and are characterized by shallow and heterogeneous soils with higher calcium (Ca) and magnesium (Mg) contents than those of other soils, and substantial leaching^4,5^. Further, these soils exhibit lower nitrogen (N) and phosphorus (P) storage than non-Karst CZs soils, and have limited plant productivity^4–7^. Plants use different leaf economic strategies to adapt to low nutrient availability^8,9^. Understanding how nutrients constrain photosynthetic capacity of mature forests in Karst CZs is a prerequisite for evaluating gross primary production and predicting the carbon cycle in these areas. The maximum carboxylation rate (*V*_cmax_) and maximum electron transport rate (*J*_max_) are proxies for photosynthetic capacity.

Leaf N and P are both essential nutrients involved in photosynthetic capacity. Photosynthetic capacity is usually positively related to leaf N because a large portion of N is invested in photosynthetic machinery^8,10,11^. Consequently, N-deficiency could reduce carboxylation capacity and electron transport rates^12^. In addition to leaf N, leaf P is one of the most important component of chemical compounds which are closely related to photosynthesis^13,14^. Consequently, P-deficiency can reduce light-use efficiency, electron transport rates^15,16^, enzyme activity in the Calvin cycle, regeneration of ribulose bisphosphate (RuBP)^17^, and the fraction of leaf N allocated to photosynthetic machinery^18^.

It is widely accepted that photosynthetic capacity at global scale is mainly controlled by leaf N and P^11,19,20^ concentrations which depend on soil nutrient status^21^. Reich and Oleksyn^22^ demonstrated that global patterns of leaf N to P ratios increase toward low latitudes and with mean temperature. Photosynthetic capacity was mainly constrained by N in temperate ecosystems^12,23^, and by P rather than N in subtropical and tropical ecosystems^24,25^. However, many previous studies reported that single-nutrient limitations or N and P co-limitation were widespread, and N and P co-limitation was more common of the two, especially in tropical ecosystems^26–29^.These studies highlighted the importance of synergistic interactions between N and P in regulating plant growth. Domingues *et al*.^30^ reported that N and P co-limited photosynthetic capacity in West Africa woodlands. Niinemets *et al*.^31^ observed that plant primary productivity in Karst grasslands (calcareous meadows) in temperate regions was co-limited by N and P due to low N and P availabilities in soil. Therefore, photosynthetic capacity in a mature subtropical Karst forest in southwestern China was expected to be co-limited by N and P.

Traditionally, primary productivity was predicted using linear relationships between photosynthetic capacity and leaf N^32^. However, this relationship can be modified by P with increasing P limitation^20^. On the basis of a cross-biome analysis of the impact of P limitation on the relationship between *A*_sat_ and N, Reich *et al*.^33^ found that the slope of *A*_sat_-N, used as an indicator of photosynthetic N use efficiency, was higher in the Arctic and temperate ecosystems at 1.59 and 1.48, respectively, than in tropical and subtropical ecosystems at 1.23 and 1.10, respectively. In a meta-analysis of global-scale data, Kattge *et al*.^10^ found that the slope of *V*_cmax_-N was flatter in tropical biomes, and the uncertainty in the relationship between *V*_cmax_ and leaf N was larger than that in other biomes. In addition, the uncertainty between *V*_cmax_ and leaf N can be decreased when considering P limitation on photosynthesis in tropical biomes^30,34^. Up till now, P limitation on photosynthetic capacity is an ongoing area of research in tropical forests^34^. However, research has yet to focus on the role of leaf P in photosynthetic capacity in a mature subtropical Karst forest, where N and P storage are limiting in soils.

In addition to N and P, other leaf mineral nutrients can modify tune the photosynthetic capacity^35,36^. Previous experiments under controlled conditions demonstrated that photosynthetic capacity can be tuned by Ca, Mg, potassium [K], and sodium [Na]. Ca ions (Ca^2+^) provide the terminal acceptor and regulate photosynthetic electron flow^37^, while Mg (Mg^2+^) and K (K^+^) ions have been implicated as light-harvesting counter-ions in thylakoids, and have opposing effects^38^. Battie-Laclau *et al*.^38^ evaluated the limitations of K and Na on *A*_sat_ in *Eucalyptus grandis*, and showed that photosynthetic capacity may be improved by supplying these two elements. However, to our knowledge, only one group has reported that *A*_sat_ was significantly and positively associated with N, P, K, Ca, and Mg, and that in five sapling tree species in the central Amazon rainforest under natural conditions^39^.

Soil quantities and storage of nutrients in Karst were much lower than those in non-Karst ecosystems due to shallow Karst soils^5,6,40^. However, Ca and Mg contents in Karst soils were higher than those in non-Karst soils^41^. The particular characteristics of Karst soils give us a unique opportunity to investigate from the point view of plant growth and economics how leaf N, P, and mineral nutrients regulate mass-based photosynthetic capacity. In this study, we selected a mature subtropical forest in the Karst CZ in southwestern China, and measured CO_2_ response curves of 63 C_3_ dominant plant species and their corresponding leaf traits (N, P, K, Ca, Mg, Na, leaf mass per area (*LMA*), and leaf thickness [*LT*]). The objective of this study was to determine whether: (1) leaf N and P co-limited photosynthetic capacity, (2) leaf mineral nutrients tune the photosynthetic capacity and if so, (3) how leaf mineral nutrients modified the relationship of photosynthetic capacity to N and photosynthetic capacity to P.

## Results

### Comparison of light-saturated net photosynthesis with the global data set

**Figure 1:**
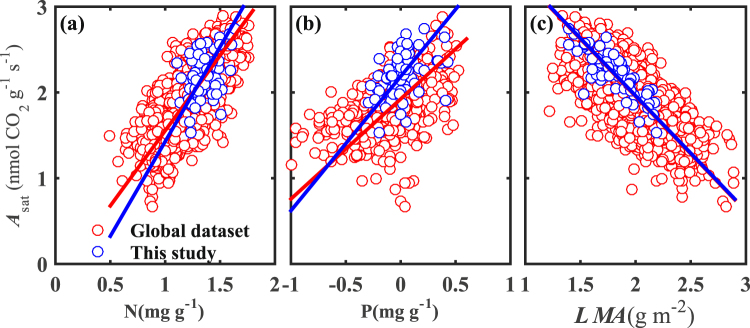
The relationships of leaf light-saturated net photosynthesis (*A*_sat_) to (**a**) leaf nitrogen (N), (**b**) phosphorus (P), and (**c**) leaf mass per area (*LMA*). Both axes are in log10 scale.

We compared the relationships of A_sat_ to leaf N, P, and *LMA* in this study with those in the global data set (Fig. 1). The averaged value of *A*_sat_ was 200.84 ± 116.63 nmol CO_2_ g^−1^ s^−1^, and ranged from 33.81 to 562.03 nmol CO_2_ g^−1^ s^−1^ (see Supplementary Table S1); this was within the normal range of the global dataset (4.65 to 778.41 nmol CO_2_ g^−1^ s^−1^)^19^.

Compared to global data set^19^, plants showed a higher *A*_sat_ for a given leaf P level in the mature subtropical forest, i.e. high photosynthetic P use efficiency. The slope of *A*_sat_-N in a standardized major axis fit was slightly but not significantly steeper (*P* = 0.333), while the intercept was slightly smaller than that in the global data set (*P* = 0.06; Fig. 1a). The slope of *A*_sat_-P was significantly steeper (*P* < 0.05), and the intercept was significantly larger than that in global data set (*P* < 0.05; Fig. 1b). The slope (*P* = 0.24) and intercept (*P* = 0.70) of *A*_sat_-*LMA* of two data sets were not significantly different (Fig. 1c).

### Relationships of *V*_cmax_ and *J*_max_ with leaf traits

We disentangled the contributions of leaf traits to photosynthetic capacity using path analysis. The Pearson correlation analysis showed that the photosynthetic capacity (*V*_cmax_, and J_max_) was positively related to leaf N, P, Mg K, and Na, and negatively related to *LT* (*P* < 0.05) (see Table S6, Figs S1–S3). Leaf N, P, Mg, and *LT* were selected using a multiple stepwise regression method (*P* < 0.1) as significant independent variables (see Table S4). Pearson correlation analysis showed that leaf N was positively related to P, negatively to *LT* (*P* < 0.05), and not related to leaf Mg (*P* > 0.05) (see Table S5). Leaf P was not related to either leaf Mg or *LT* (*P* > 0.05). Leaf Mg was not related to *LT* (*P* > 0.05). These results indicated that leaf N, P, Mg, and *LT* had the potential to alter photosynthetic capacity directly, and leaf N was correlated with leaf P and *LT*.

**Figure 2:**
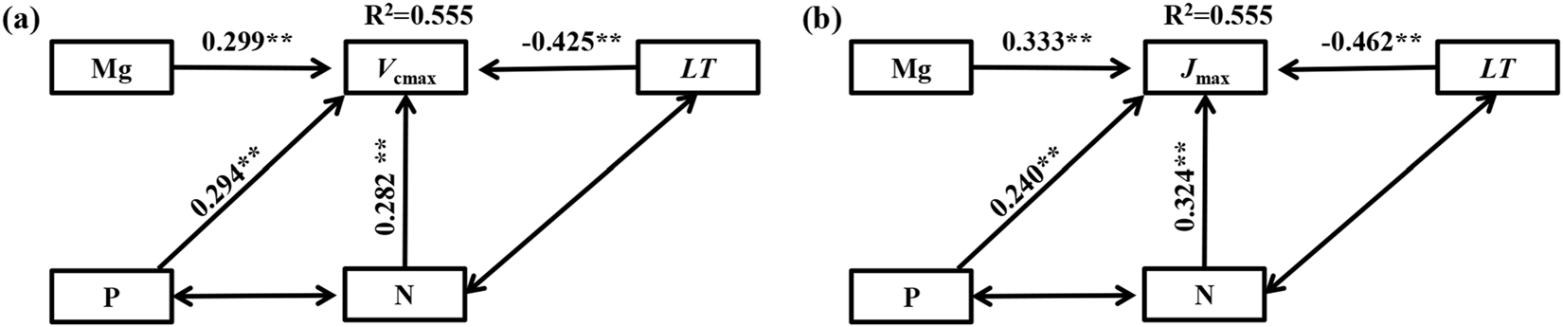
The direct and indirect causality of leaf nitrogen (N), magnesium (Mg), and leaf thickness (*LT*) on (**a**) maximum carboxylation rate (*V*_cmax_) and (**b**) maximum electron transport rate (*J*_max_). One way arrow indicates causality relationship between two variables; Two-way arrows represent correlated relationship between two variables. **P < 0.05, *P < 0.1. Results of model fitting: (a) χ^2^ = 0.486, d.f. = 4, *P* = 0.746, AIC = 23.944; (b) χ^2^ = 0.486, d.f. = 4, *P* = 0.746, AIC = 23.944.

The causal relationships and relative contributions of leaf N, P, Mg, and *LT* to *V*_cmax_ and *J*_max_ were presented in Fig. 2. The models explain 55.5% and 55.5% of the variation in *V*_cmax_ and *J*_max_, respectively. The total contribution of leaf N, P, Mg, and *LT* to *V*_cmax_ was 0.282, 0.294, 0.299, and −0.425, and to *J*_max_, it was 0.324, 0.240, 0.333, and −0.462, respectively. These results indicated that photosynthetic capacity was influenced by leaf N, P, Mg, and *LT*.

### Relationships of photosynthetic N and P use efficiency to leaf traits

**Figure 3:**
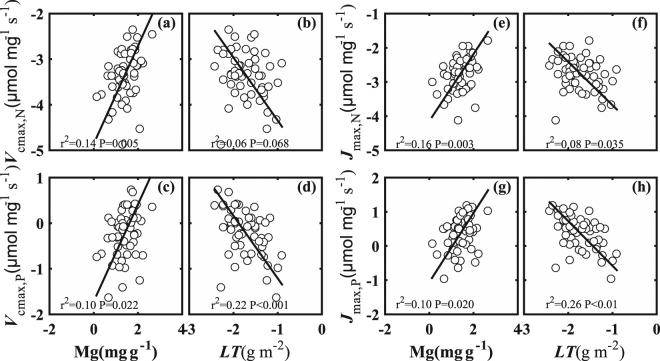
Log-log plots of the ratio of maximum carboxylation rate (*V*_cmax_) to leaf nitrogen (N) (*V*_cmax,N_) in relation to (**a**) leaf magnesium (Mg) and (**b**) leaf thickness (*LT*). Log-log plots of the ratio of *V*_cmax_ to P (*V*_cmax,P_) in relation to (**c**) Mg and (**d**) LMA. Log-log plots of the ratio of maximum electron transport rate (*J*_max_) to N (*J*_max,N_) in relation to (**e**) Mg and (**f**) *LT*. Log-log plots of the ratio of *J*_max_ to P (*J*_max,P_) in relation to (**g**) Mg and (**h**) *LT*.

As a whole, photosynthetic N and P use efficiencies were promoted by leaf Mg but limited by *LT* (Fig. 3). The effect of Mg on photosynthetic N use efficiency was similar to that of photosynthetic P use efficiency. The effect of *LT* on photosynthetic P use efficiency was less than that on photosynthetic N use efficiency. No relationship was found between leaf Mg and *LT* (*P* > 0.05) (see Table S5).

The effect of Mg on photosynthetic N use efficiency was similar to photosynthetic P use efficiency. The *V*_cmax,N_ (*R*^2^ = 0.14, *P* < 0.05), *V*_cmax,P_ (*R*^2^ = 0.10, *P* < 0.05), *J*_max,N_ (*R*^2^ = 0.16, *P* < 0.05), and *J*_max,P_ (*R*^2^ = 0.10, *P* < 0.05) were positively related to Mg. The slopes of *V*_cmax,N_-Mg (1.07) and *V*_cmax,P_-Mg (1.06) were larger than those of *J*_max,N_-Mg (0.98) and *J*_max,P_-Mg (1.01). These results showed that the photosynthetic N and P use efficiency was positively correlated with leaf Mg.

The effect of *LT* on photosynthetic P use efficiency was less than that on photosynthetic N use efficiency. The *V*_cmax,N_ (*R*^2^ = 0.06, *P* = 0.068), *V*_cmax,P_ (*R*^2^ = 0.22, *P* < 0.05), *J*_max,N_ (*R*^2^ = 0.08, *P* < 0.05), and *J*_max,P_ (*R*^2^ = 0.26, *P* < 0.05) showed a significant negative relationship with *LT*. The slopes of *V*_cmax,N_-*LT* (−1.38) and *V*_cmax,P_-*LT* (−1.37) were smaller than those of *J*_max,N_-*LT* (−1.27) and *J*_max,P_-*LT* (−1.31).

## Discussion

### Argument for mass-based vs. area-based photosynthetic capacity

The ‘leaf economic spectrum’ of traits has been described by Wright *et al*.^11^, who demonstrated that the mass-based photosynthetic capacity was positively related to mass-based leaf N and P content, and negatively related to *LMA* and leaf lifespan. Recently, the biological significance of the ‘leaf economic spectrum’ has become the focus of the debate. Lloyd *et al*.^42^ and Osnas *et al*.^43^ suggested that these correlations were driven by the variation in *LMA*, which determined the ratio of structural to metabolic components of the leaves. They thought that the photosynthetic parameters and the associated leaf nutrient traits should be expressed from the viewpoint of photosynthetic physiology on an area-basis. However, Westoby *et al*.^44^ and Poorter *et al*.^45^ emphasized the critical role of mass-based photosynthetic parameters and the corresponding leaf nutrient traits in plant growth and economics. They thought the mass-based leaf trait was a way to express the difference among species in costs and returns per unit investment.

In this study, we mainly investigated how leaf N, P, and mineral nutrients regulated mass-based photosynthetic capacity from the viewpoint of plant growth and economics. In addition, we also presented the relationship between area-based photosynthetic capacity and associated leaf traits in the Supplementary Tables S1 an S2, and discussed it below, where relevant.

### Leaf N and P co-limited photosynthetic capacity

Leaf N and P are generally the major growth-limiting nutrients for plant communities when key physiological processes are considered^46^. The averaged leaf N content in this study was 23.39 ± 6.72 mg g^−1^ (see Table S7), larger than that reported by Reich & Oleksyn^22^ for 2151 plant species (20.1 mg g^−1^), and by Maire *et al*.^19^ for 1658 plant species (19.49 ± 9.30 mg g^−1^). The averaged leaf P content in this study was 1.11 ± 0.50 mg g^−1^ (see Table S7), 37% lower than the global average reported by Reich & Oleksyn^22^ for 923 plant species (1.77 mg g^−1^), and nearly identical to that reported by Maire *et al*.^19^ for 522 plant species (1.03 ± 0.65 mg g^−1^). Note that the data set of leaf P in Fig. 1b was reported by Maire *et al*.^19^, and leaf P was associated with *A*_sat_. The averaged leaf N:P in this study was 23.34 ± 7.81, indicating P limitation^22^.

The importance of synergistic interactions between N and P in regulating plant growth has been reported in many previous studies^26–29^. Consistent with Karst grassland^31^ and West Africa woodlands^30^, photosynthetic capacity was co-limited by N and P in this study (Fig. 2). The seemingly contradictory results can be explained by leaf economy and the differences in allocation strategies of leaf N and P.

There was a trade-off between leaf N allocation to metabolic N and structural N as means of adaptation to the limited nutrient conditions^47,48^. When nutrient availability was low, the fraction of leaf N partitioned to cell walls was greater, thereby *LMA* was high and rates of photosynthesis decreased^49,50^. The range of variation in *LMA* (24.73–154.61 g m^−2^) in this study was larger than that for subtropical non-Karst forest (37.08–142.32 g m^−2^)^51^. *LT* was negatively related to leaf N and photosynthetic capacity (see Tables S6). In addition, photosynthesis and its N use efficiency increased with a decrease in N allocation to leaf non-photosynthesis^8,47^. In this study, *A*_sat_ and photosynthetic capacity were negatively related to *LT* (see Table S6). Photosynthetic N use efficiency (slope of *A*_sat_-N) in this study was higher than that in other tropical ecosystems^33^. On the other hand, no relationship was found between area-based photosynthetic capacity and the associated leaf N (see Table S3). These results may indicate that a trade-off existed between leaf N allocation into metabolic and structural N in this mature Karst forest.

However, there was no apparent trade-off between leaf P allocation into metabolic P and structural P^9^. Leaf P was preferentially allocated to photosynthetic cells in P-limited conditions^52^. The fraction of P in structural tissues was one order of magnitude lower than that of N^53^. With decreasing soil P availability, the *LMA* of tropical trees increased, and leaf P content decreased; however, tropical trees can maintain high photosynthetic P use efficiency without increasing P allocation into structural tissues^9^. In this study, mass-based leaf P was not related to *LT* (*P* > 0.05) (see Table S5), and positively related to mass-based photosynthetic capacity (*P* < 0.05) (see Table S6). Photosynthetic P use efficiency in Karst plants was higher than that in other tropical ecosystems (Fig. 1b). In addition, area-based photosynthetic capacity was weakly related to leaf P (*P* < 0.05) (see Table S3). Based on the results mentioned above, we suggested that no trade-off existed between leaf P allocation into metabolic P and structural P in this mature Karst forest.

It is commonly assumed that leaf N:P ratio is often used as a proxy for nutrient limitation. Leaf N:P ratio of <14 indicates that N is the limiting factor, while >16 that P is limiting^22^. However, these ratios differ when applied to different ecosystems. For example, productivity of desert shrublands was limited by P at N:P of 5–10^54^, while productivity of invasive species was limited by N at N:P >40^55^. Productivity of Karst grassland was co-limited by N and P at N:P of 5.6–7.5^31^. The different leaf N and P allocation strategies was the main reason for the high N:P in this mature Karst forest.

### Leaf Mg tuned photosynthetic capacity

The contribution of leaf Mg to *V*_cmax_ and *J*_max_ was 0.299 and 0.333, respectively (Fig. 2), and photosynthetic N and P use efficiencies were positively related to Mg (Fig. 3). This was consistent with the results reported by Mendes & Marenco^39^ for tropical saplings. However, photosynthetic capacity of West Africa woodlands was not related to Mg, Ca, K etc.^30^. The averaged leaf Mg content was 4.61 ± 2.39 mg g^−1^, which was higher than that in non-Karst tropical and temperate forests^56^. We speculate that the photosynthetic capacity might be tuned by leaf Mg via enhancing photosynthetic N and P efficiency; a possible mechanism for this may involve the key role which leaf Mg plays in photosynthesis^57^.

**Figure 4:**
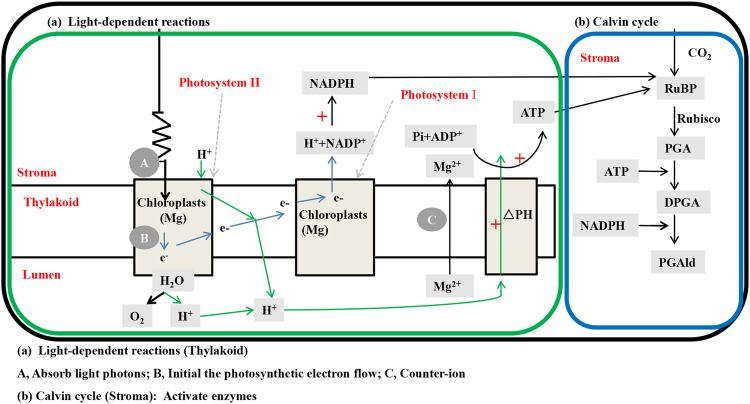
Roles of Mg in photosynthetic processes: **(a)** light-dependent reactions (A, absorb light photons^59–61^; B, initial the photosynthetic electron flow^62,63^; C, Counter-ion^64,66^) and (**b**) Calvin cycle stages (Part 2: active enzymes^66–68^). The blue arrows indicate the electron flow between photosystem II and photosystem I. The moving of hydrogen ions (H^+^) is indicated by the green arrows. The red “+” represents the positive effect of Mg on biochemical and physiological processes. ATP, adenosine triphosphate; ADP, adenosine diphosphate; NADPH, nicotinamide adenine dinucleotide phosphate; RuBP, ribulose 1,5-biphosphate; Rubisco, ribulose-1,5-bisphosphate carboxylase/oxygenase; Ribulose bisphosphate carboxylase oxygenase; PGA, 3-phosphoglyceric acid; DPGA, 1,3-diphosphoglycerate; PGAld, glyceraladehyde-3-phosphate. Figure 4a was modified from Alexander N. Tikhonov^64^. Republished with permission of Springer Science and Bus Media B V, from Photosynthesis Research, Alexander N. Tikhonov, Volume 116, issue 2–3, pp 511–534, 2013; permission conveyed through Copyright Clearance Center, Inc.

During the light-dependent reactions and the Calvin-cycle stages of photosynthesis, Mg is involved in three key biochemical processes (Fig. 4). First, as a light-dependent reaction, the chlorophyll molecule, which is composed of a central Mg ion surrounded by a group of atoms, is catalyzed by Mg^56,58^ (Fig. 4a). Neuhaus *et al*.^59^ and Jezek *et al*.^60^ have reported that Mg fertilizer can increase the concentration of chlorophyll, thus enhancing light harvesting efficiency^61^ and electron transport rates^38,62,63^; then, formation rates of nicotinamide adenine dinucleotide phosphate (NADPH) can also increase because NADP^+^ is the terminal acceptor of electron transport^64^.

Second, Mg can also promote the synthesis of adenosine triphosphate (ATP)^65^ (Fig. 4a). During electron transport, protons are pumped from the stroma into the thylakoid lumen, thus generating a proton (H^+^) gradient^57,66^ driving the synthesis of ATP^38^. When protons are pumped into the thylakoid lumen, Mg^2+^ is transported into the stroma from the lumen as a counter-ion^64^. The stimulating role of Mg in the H^+^ pump has been confirmed by Kana and Govindjee^38^.

Third, Mg is a cofactor and allosteric modulator for enzymes, and regulates the Calvin cycle by activating many enzymes^66^ (Fig. 4b). For example, ribulose-1,5-bisphosphate carboxylase (Rubisco) was activated when incubated with CO_2_ and Mg^2+ ^^67^. Also, Pradel *et al*.^68^ showed that the concentration of fructose 1,6-bisphosphatase increased with increasing Mg.

However, these results reported by previous studies were obtained under controlled conditions using low Mg supply. Considering the important role of Mg in photosynthesis in low-nutrient ecosystems^39^, there is an urgent need to explore how leaf Mg tunes photosynthetic capacity under natural conditions, especially in nutrient-poor soils.

## Conclusions

Our results revealed that the photosynthetic capacity of Karst plants was co-constrained by N, P, Mg, and *LT*. Our analysis indicated that nutrient interactions were complex in biochemical and physiological processes. We propose that the accurate prediction of *V*_cmax_ and *J*_max_ in a mature subtropical forest with high Ca and Mg should take into consideration not only the role of N and P but also of other mineral nutrients.

## Methods

### Site information

This research was conducted in a mature subtropical forest (26°14′48″N, 105°45′51″E; elevation, 1460 m) located in Puding County, Guizhou Province, in a Karst critical zone in southwestern China. The climate is subtropical monsoonal, with a mean annual precipitation of 1255 mm and a mean annual air temperature of 15.1 °C^69^.

Soils in this region were mainly formed by limestone and dolomite^70^. In this study, the (total and available) soil N and P content (see Table S7) was similar to that of other Karst soils in southwestern China^71^, but higher than that of non-Karst soils^72^. However, soil quantities (16.04~61.89 kg m^−2^) and nutrient storage (see Supplementary Table S7) were much lower than those of non-Karst ecosystem^5,6^, because of the shallow and heterogeneous **s**oil layer (2–50 cm)^73–76^.

Vegetation type is a mature mixed evergreen and broad-leaved deciduous forest which is remarkably different from the non-karst forest in this region (subtropical evergreen broad-leaved)^5^. The dominant species include *Itea yunnanensis* Franch, *Carpinus pubescens* Burk., and *Lithocarpus confinis* Huang *et al*. (see Supplementary Table S1). Mean content of leaf N, P, Ca, Mg, Na, and K can be found in Table S7. The aboveground carbon stock in mature Karst forest in southwestern China was lower (70.3–142.2 Mg ha^−1^) than that in subtropical evergreen broad-leaved forests growing in non-Karst regions^4^. Further, the aboveground carbon stock in this study was higher than that of mature Karst forest in Mexico^7^, and lower than that of mature Mediterranean forest in Italy^77^; these differences were probably due to different thicknesses of the soil, and the amount of precipitation.

### Gas exchange measurements

Leaf gas exchange was measured from July to August, 2016 using a portable photosynthesis system. This system consisted of an infrared gas analyzer (Li-Cor 6400; Li-Cor Bio Sciences, Lincoln, NE), an artificial light source (6400–02B red/blue LED light source; Li-Cor Bio Sciences), a CO_2_ injection system with pure CO_2_, and a CO_2_ absorbent system with a buffer bottle which supplied stable air flow without CO_2_. Three individuals per species were collected and measured, with a total of 189 individuals from 63 dominant species (see Supplementary Table S1) in the mature forest. Branches with sun leaves were excised from the upper part of the crown using a lopper (6 m), and immediately stored in a bucket; later, branches were snipped under water with shears to maintain xylem water continuity^30^. Prior to gas exchange measurements, branches were kept at 25°C for 30 min; then, a fully-expanded, mature leaf was induced for 30 minutes at a saturating light density (1500 μmol m^−2^ s^−1^).

The CO_2_ response curves (*A*-*C*i curve) of light-saturated photosynthesis were determined following procedural guidelines^78^. In brief, CO_2_ concentrations inside the chamber varied from 50 to 1800 μmol mol^−1^(400, 300, 200, 100, 50, 400, 600, 800, 1200, 1400, 1600 and 1800 μmol mol^−1^). CO_2_ concentrations were controlled by a CO_2_ injector system. Photosynthetic photon flux density was set to 1500 μmol m^−2^ s^−1^, which was controlled by an artificial light source. The leaf temperature was controlled by the conditioning the block temperature to 25 °C, and the vapor pressure deficit was maintained at ambient condition. Flow rate in the cuvette was set to 500 mL min^−1^. The cuvette was sealed with plasticine to prevent leakage.

### Leaf trait analyses

Immediately after the measurements of leaf gas exchange, leaf area (m^−2^), fresh mass (mg), and *LT* (mm) were measured. After that, leaves were oven dried at 40 °C for 48 h, and dry mass (mg) was determined. The *LMA* (g m^−2^) was calculated by dividing the corresponding dry mass by leaf area. Thereafter, dried leaves were ground to a powder for nutrient analysis. Mass-based leaf carbon (C) and N contents (mg g^−1^) were determined by elemental analysis (EURO EA CHNSO Analyser; HEKAtech GmbH, Wegberg, Germany). Mass-based leaf P, Ca, Mg, K, and Na contents (mg g^−1^) were measured using inductively-coupled plasma-optical emission spectrometry (Optima 5300 DV; Perkin Elmer, Waltham, MA). All auxiliary datasets were presented in Supplementary Table S2; for more related information, see He *et al*.^79^.

### Response curve analyses

Area-based *A*_sat_ (μmol CO_2_ m^−2^ s^−1^) under saturating light (1500 μmol m^−2^ s^−1^) and CO_2_ concentration (400 μmol mol^−1^) was extracted from the *A*-*C*i curves^30^. Area-based *V*_cmax_ and *J*_max_ (μmol CO_2_ m^−2^ s^−1^) were estimated using the Farquhar biochemical model^80,81^. We used the curve-fitting routine developed by Domingues *et al*.^30^. The enzymatic kinetic constants used in the curve-fitting routine were taken from von Caemmerer^81^. Mesophyll conductance was not estimated, but rather assumed to be infinite. Therefore, *V*_cmax_ and *J*_max_ were determined based on the intercellular CO_2_ concentration. To compare with existing databases, calculated *V*_cmax_ and *J*_max_ were standardized to 25 °C^82^.

Mass-based *A*_sat_, *V*_cmax_, and *J*_max_ were calculated by dividing area-based *A*_sat_, *V*_cmax_, and *J*_max_ by the corresponding *LMA*. For photosynthetic N use efficiency (μmol CO_2_ mg^−1^ s^−1^), we defined ratios as *V*_cmax_ to N (*V*_cmax__,__N_), and *J*_max_ to N (*J*_cmax__,__N_). We defined photosynthetic P use efficiency (μmol CO_2_ mg^−1^ s^−1^) as the ratio of *V*_cmax_ to P (*V*_cmax__,__P_), and of *J*_max_ to P (*J*_cmax__,__P_).

The relationships between area-based leaf nutrients and photosynthetic capacity are shown in Supplementary Table S3. Area-based leaf nutrients were the product of mass-based leaf nutrient content and *LMA*.

### Statistical analysis

Contributions of leaf traits to *V*_cmax_ and *J*_max_ were determined by path analysis^83,84^. In brief, the advantage of path analysis is to disentangle the causality between variables, and to quantify contributions of independent variables to dependent variable when a prior causal or correlative relationship among variables is known. Path coefficient is a statistic used to represent the causality of the related variables, and is a normalized partial regression coefficient. The contributions of independent variables to dependent variables were represented by path coefficients. A positive value of a path coefficient represented positive contribution, and vice versa. Total contribution of one of the independent variables to the dependent variable was the sum of the direct and indirect path coefficients. The proportion of variance explained was represented by R^2^. The model had a good fit when 0 ⩽ χ^2^ ⩽ 2 and 0.05 < P ⩽ 1.

The hypothesized causal relationships between photosynthetic capacity and leaf traits were developed as follows. We assumed that the photosynthetic capacity was regulated by leaf N, P, Ca, Mg, Na, K, and *LT*. Pearson correlation analysis showed that photosynthetic capacity (*V*_cmax_, and J_max_) was positively related to leaf N, P, Mg, K, and Na, and negatively related to *LT* (*P* < 0.05) (see Supplementary Table S6, Figs S1–S3). A stepwise multiple regression analysis was performed to select significant independent variables among leaf traits. *J*_cmax_ was co-regulated by leaf N, P, Mg, and *LT*; however, *V*_cmax_ was co-regulated by leaf N, Mg, and *LT* at the 0.05 level. *V*_cmax_ and *J*_max_ were co-regulated by leaf N, P, Mg, and *LT* at the 0.1 level. In this study, leaf N, P, Mg, and *LT* were selected as significant independent variables using multiple stepwise regression method (*P* < 0.1) (see Supplementary Table S4). Pearson correlation analysis showed that leaf N was positively related to P, negatively to *LT*, and not related to leaf Mg. Leaf P was not related to leaf Mg or to *LT* (*P* > 0.05). Leaf Mg was not related to *LT* (*P* > 0.05). According to these results, we proposed that leaf N, P, Mg, and *LT* had potential to alter photosynthetic capacity directly, and leaf N was correlated with leaf P and with *LT*. Path analyses were performed using AMOs 23.0 (Amos Development CO., Greene, Maine, USA).

Standardized major axis (SMA) regression fit was applied to compare the slope and intercept of *A*_sat_-N, *A*_sat_-P and *A*_sat_-*LMA* in this study with the global dataset^19^. The relationships between photosynthetic N and P use efficiency (*V*_cmax__,__N_, *J*_cmax__,__N_, *V*_cmax__,__P_ and *J*_cmax__,__P_) and main contributors of *V*_cmax_ and *J*_max_ (Mg and *TL*) were determined by linear regression of least square method.

## Electronic supplementary material

Supplementary Information
