# Supplementary material for: Co-regulation of photosynthetic capacity by nitrogen, phosphorus and magnesium in a subtropical Karst forest in China
Source: Sci Rep. 2018 May 9;8:7406. doi: 10.1038/s41598-018-25839-1 (PMC5943327; doi:10.1038/s41598-018-25839-1)
Supplement: Supplementary file 1 — Supplementary Information [file 41598_2018_25839_MOESM1_ESM.pdf]

Supplementary Information

Co-regulation of photosynthetic capacity by nitrogen, phosphorus and magnesium in a subtropical Karst forest in China

Jing Wang[1,2,3]; Xuefa Wen[1,2*], Xinyu Zhang[1,2*], Shenggong Li[1,2], Da-Yong Zhang[3]

Contents of supplementary information

Supplementary Table S1. The area-based and dry mass-based light saturated assimilation rates (A_{sat}), maximum rate of photosynthetic carboxylation (V_{cmax}), and maximum rate of photosynthetic electron transport (J_{max}) of dominant species (n=63) selected from a subtropical mature forest in the Karst critical zone in Southwestern China.

Supplementary Table S2. The averaged leaf nutrient concentrations, leaf mass per area (LMA), and leaf thickness (*LT*).

Supplementary Table S3. Pearson correlations of the area-based photosynthetic capacity (A_{sat}, V_{cmax}, and J_{max}) with leaf nutrient contents and leaf thickness (*LT*). A_{sat}: the light-saturated net photosynthesis; V_{cmax}: the maximum carboxylation rate; J_{max}: the maximum electron transport rate.

Supplementary Table S4. Coefficients of multivariate stepwise regression. V_{cmax}: the maximum carboxylation rate; J_{max}: the maximum electron transport rate.

Supplementary Table S5. Pearson correlations of mass-based leaf nutrient contents and leaf thickness (*LT*).

Supplementary Table S6. Pearson correlations of mass-based photosynthetic capacity (V_{cmax} and J_{max}) with leaf nutrient contents and leaf thickness (*LT*). A_{sat}: the light-saturated net photosynthesis; V_{cmax}: the maximum carboxylation rate; J_{max}: the maximum electron transport rate.

Supplementary Table S7. Characteristics of soil and leaf in this study site. TN, Total nitrogen; TP, total phosphorus; TCa, total calcium; TMg, total magnesium; TK, total potassium; TNa, total sodium; AN, available soil nitrogen; AP, available soil phosphorus.

Figure S1. Relationships between of the A_{sat} (nmol g^{-1} s^{-1}) and leaf traits. A_{sat}, the light-saturated net photosynthesis; *LT*, leaf thickness (mm); Leaf nutrients (mg g^{-1}).

Figure S2. Relationships between of V_{cmax} (μmol g^{-1} s^{-1}) and leaf traits. V_{cmax}, the maximum carboxylation rate; *LT*, leaf thickness (mm); Leaf nutrients (mg g^{-1}).

Figure S3. Relationships between of J_{max} (μmol g^{-1} s^{-1}) with leaf traits(mg g^{-1}). J_{max}, the maximum electron transport rate; *LT*, leaf thickness (mm); Leaf nutrients (mg g^{-1}).

Supplementary Table S1. The area-based and dry mass-based light saturated assimilation rates (A_{sat}), maximum rate of photosynthetic carboxylation (V_{cmax}), and maximum rate of photosynthetic electron transport (J_{max}) of dominant species (n=63) selected from a subtropical mature forest in the Karst critical zone in Southwestern China.

Species	A_{sat} (umol m^{-2} s^{-1})	V_{cmax} (umol m^{-2} s^{-1})	J_{max} (umol m^{-2} s^{-1})	A_{sat} (nmol g^{-1} s^{-1})	*V*cmax (nmol g^{-1} s^{-}1)	*J*max (nmol g^{-1} s^{-}1)
Ligustrum lucidum Ait.	11.73±0.91	54.39±3.72	93.71±10.54	149.43±11.56	692.68±47.34	1193.47±7.33
Rhamnus leptophylla Schneid.	12.53±0.4	55.5±3.42	107.27±5.29	187.11±6.03	828.61±51.07	1601.41±7.11
Lindera communis Hemsl.	9.09±0.38	39.71±3.5	60.11±1.52	93.12±3.86	406.82±35.84	615.81±0.82
Itea yunnanensis Franch	6.91±0.77	35.61±3.75	80.67±3.84	72.82±8.08	375.23±39.55	850.12±1.84
Pittosporum brevicalyx (Oliv.) Gagnep	7.36±0.77	39.39±3.6	79.46±11.27	112.12±11.75	600.42±54.81	1211.33±15.38
Litsea rubescens Lec.	11.9±1.54	63.74±6.8	125.42±8.59	320.8±41.5	1718.29±183.39	3381.03±28.75
Broussonetia papyifera (Linn.) L'Hert. ex Vent.	15.67±1.55	70.85±10.45	112.86±7.02	236.41±23.39	1069.04±157.75	1703.03±15.13
Machilus microcarpa Hemsl.	7.69±2.05	37.06±6.33	64.75±5.64	171.3±45.77	825.6±140.89	1442.28±12.34
Rhus chinensis Mill.	14.9±0.3	69.31±0.66	103.12±22.19	187.6±3.78	872.64±8.28	1298.31±25.99
Melia azedarach L.	14.13±1.44	73.09±6.32	121.38±13.43	146.68±14.91	758.53±65.55	1259.77±14.64
Populus × canadensis Moench.	9.64±1.34	42.39±4.47	70.86±5.69	80.21±11.11	352.67±37.22	589.57±2.5
Camptotheca acuminata Decne.	10.16±1.81	48.57±1.89	91.35±0.65	169.97±30.25	812.19±31.56	1527.7±1.01
*Cinnamomum bodinier*i Levl.	13.07±2.26	65.65±3.81	122.71±13.1	143.16±24.75	719.24±41.79	1344.44±6.79
Catalpa ovata G. Don	23.67±1.33	99.56±10.68	147.64±20.04	441.09±24.82	1855.61±199.12	2751.74±40.47
Toona sinensis (A. Juss.) Roem.	9±1.94	42.35±7.07	79.13±3.01	361.72±78.08	1702.16±284.28	3180.52±8.32
Sapium sebiferum (Linn.) Roxb.	14.47±2.74	65.21±6.74	98.95±7.5	192.57±36.51	867.99±89.72	1317.17±9.19
Alangium chinense (Lour.) Harms	11.16±0.62	57.84±5.56	104.6±9.08	127.44±7.13	660.55±63.51	1194.53±11.33
Cladrastis platycarpa (Maxim.) Makino	11.55±1.62	59.29±5.66	116.21±10.78	234.66±32.92	1204.17±114.89	2360.25±12.66
Ulmus pumila L.	10.77±1.19	43.17±12.11	57.84±15.4	157.42±17.34	630.83±176.98	845.31±17.61
Ilex macrocarpa Oliv.	6.29±0.3	38.75±0.97	90.17±2.85	76.28±3.61	469.95±11.76	1093.47±1.98
Ziziphus jujuba Mill. var. *spinosa* (Bunge) Hu ex H. F. Chow	15.17±0.55	62.11±2.07	96.58±4.97	107.03±3.89	438.29±14.58	681.54±4.63

Vitex canescens Kurz	10.7±1.91	56.15±5.67	84.21±5.02	219.85±39.2	1153.67±116.47	1730.17±8.59
Eriobotrya japonica (Thunb.) Lindl.	10.87±0.06	51.22±2.77	94.13±4.91	122.28±0.65	576.33±31.15	1059.2±4.46
Morus alba L.	13.3±2.72	64.18±11.74	102.4±17.98	484.69±99.2	2338.8±427.94	3731.84±42.67
Prunus salicina Lindl.	12.93±1.12	54.66±5.34	105.05±12.44	242.64±20.92	1025.36±100.12	1970.72±21.99
Eucommia ulmoides Oliver	10.19±0.71	48.19±9.4	109.39±6.54	181.05±12.58	856.55±167.1	1944.29±15.72
Platycarya strobilacea Sieb. et Zucc.	11.27±1	44.09±3.13	92.09±4.45	246.52±21.92	964.67±68.56	2014.9±13.88
Kalopanax septemlobus (Thunb.) Koidz.	11.47±2.27	62.99±13.46	95.25±10.96	340.74±67.31	1870.81±399.87	2828.86±30.61
Zanthoxylum armatum DC.	13.13±2.86	72.76±4.92	123.69±30.27	100.15±21.86	555.12±37.55	943.64±18.91
Pyrus calleryana	8.68±0.85	40.78±2.17	71.86±5.48	110.6±10.88	519.38±27.6	915.21±7.18
Amygdalus persica L. var.	16.45±1.06	73.36±1.56	117.6±3.28	222.4±14.38	992.1±21.06	1590.33±2.7
Euonymus meaackii Rupr.	9.74±0.16	55.27±2.57	117.33±6.66	81.07±1.35	459.84±21.35	976.2±3.96
Zanthoxylum ovalifolium Wight	9.33±1.23	56.71±3.05	114.9±5.8	104.18±13.69	632.97±34	1282.49±3.64
Cerasus scopulorum (Koehne) Yu *et* Li	13.47±0.83	55.01±3.66	106.21±4.67	178.42±11.03	728.76±48.5	1407.16±4.57
Carpinus pubescens Burk.	5.72±0.66	26.8±0	52.28±0	145.71±16.93	682.59±31.06	1331.75±8.09
Lithocarpus confinis Huang	11.28±1.78	53.98±4.59	81.33±4.09	76.97±12.15	368.34±31.35	555±2.14
Celtis sinensis Pers.	10.35±2.56	47.8±11.87	87.64±18.25	82.92±20.46	382.82±95.07	701.95±15.3
Evodia rutaecarpa (Juss.) Benth.	9.79±0.97	57.9±6.55	111.13±9.44	74.83±7.42	442.67±50.11	849.7±5.73
Machilus cavaleriei Levl.	8.38±0.49	37.17±1.63	71.78±10.95	65.07±3.78	288.74±12.65	557.54±7.39
Diospyros kaki Thunb. var. *silvestris* Makino	10.74±2.04	47.24±5.17	81.13±3.51	123.92±23.59	545.1±59.67	936.14±5.24
Rubus inopertus (Diels) Focke	14.5±2.23	59.77±7.87	95.34±9.07	380.28±58.59	1567.48±206.28	2500.36±17.52
Coriaria nepalensis Wall.	13.46±0.8	65.34±4.17	119.16±5.28	104.38±6.18	506.58±32.3	923.89±6.83
Celastrus orbiculatus Thunb.	5.23±0.27	28.25±2.57	61.19±4.98	33.81±1.73	182.69±16.59	395.74±2.7
Wikstroemia scytophylla Diels	12.51±2.89	66.31±7.24	108.41±10.2	364.7±84.11	1932.99±211	3160.37±28.24
Viburnum foetidum Wall. var. *ceanothoides* (C. H. Wright) Hand.-Mazz.	9.02±0.99	39.43±5.56	63.86±8.45	306.03±33.48	1338.25±188.67	2167.54±14.42
Hedera nepalensis K. Koch	6.66±2.52	31.91±9.37	54.01±27.19	139.05±52.72	666.33±195.64	1127.71±48.5

var. *sinensis* (Tobl.) Rehd.						
Debregeasia longifolia (Burm. f.) Wedd.	16.23±1.16	69.84±7.83	107.47±13.75	231.79±16.55	997.18±111.81	1534.51±16.88
Rubus parvifolius L.	16.33±0.87	76.48±11.57	111.05±9.32	287.08±15.36	1344.18±203.26	1951.78±12.97
Rosa roxbunghii	12.1±0.7	52.38±2.92	102.72±5.91	95.28±5.51	412.49±23.02	808.87±5.25
Mallotus repandus (Willd.) Muell. Arg.	7.09±1.3	36.97±8	70.24±15.58	160.51±29.47	836.61±181.08	1589.29±26.93
Mahonia bealei (Fort.) Carr.	4.83±0.32	23.67±1.11	45.9±2.32	45.11±2.95	221.22±10.4	428.98±2.16
Fallopia multiflora (Thunb.) Harald.	16.63±4.23	78.54±8.48	153.18±32.54	352.5±89.72	1664.4±179.74	3246.29±63.99
Conyza canadensis (L.) Cronq.	13.9±0.35	60.34±2.54	97.64±4.62	562.03±14.01	2439.93±102.77	3948.1±5.36
Ipomoea batatas (L.) Lam.	17.37±0.85	71.7±1.28	122.29±4.51	276.31±13.53	1140.81±20.43	1945.76±3.71
Senecio scandens Buch.-Ham. ex D. Don	12.27±1.23	55.9±5.2	97.48±6.98	227.97±22.94	1038.87±96.68	1811.57±10.95
Vitis piasezkii Maxim.	12.53±1.37	59.12±3.85	103.33±11.85	366.25±39.89	1727.55±112.49	3019.52±31.39
Clematis urophylla Franch.	8.1±1.06	35.63±1.05	76.61±1.57	55.45±7.28	244.04±7.17	524.65±0.44
Bauhinia glauca (Wall. ex Benth.) Benth.	7.84±1.12	35.41±4.54	68.07±3.73	157.3±22.56	710.65±91.17	1366.2±1.01
Caesalpinia decapetala (Roth) Alston	11.27±1.42	52.8±4.24	94.84±29.96	198.3±24.91	929.31±74.66	1669.13±49.22
Paederia scandens (Lour.) Merr.	10.14±0.66	42.62±4.37	69.68±9.52	250.6±16.33	1053.64±108.02	1722.51±21.09
Cyclosorus parasiticus (L.) Farwell.	7.59±1.3	35.4±4.2	72.38±8.91	185.74±31.71	866.4±102.71	1771.27±24.29
Cyrtomium fortunei J. Sm.	3.24±0.47	16.72±3	36.58±6.56	95.64±13.76	493.49±88.57	1079.82±24.58
Pteris vittata L.	9±2.95	49.2±12	84.99±29.55	155.39±50.97	849.69±207.18	1467.88±50.61

Table S2. The averaged leaf nutrient concentrations, leaf mass per area (LMA), and leaf thickness (*LT*).

Species	N (mg g^{-1})	P (mg g^{-1})	Ca (mg g^{-1})	Mg (mg g^{-1})	K (mg g^{-1})	Na (mg g^{-1})	LMA (mg m^{-2})	*LT* (mm)
Ligustrum lucidum Ait.	1.328	0.771	15.244	5.170	9.658	0.036	78.519	0.222
Rhamnus leptophylla Schneid.	2.339	0.826	31.390	3.635	9.484	0.037	66.983	0.140
Lindera communis Hemsl.	1.739	0.799	6.355	1.415	7.025	0.031	97.613	0.203
Itea yunnanensis Franch	1.705	0.789	19.636	1.125	8.501	0.032	94.892	0.267
Pittosporum brevicalyx (Oliv.) Gagnep	1.521	0.615	16.309	3.802	13.883	0.198	65.598	0.127
Litsea rubescens Lec.	2.559	1.179	10.181	6.394	12.255	0.044	37.095	0.108
Broussonetia papyifera (Linn.) L'Hert. ex Vent.	2.592	2.163	31.759	9.203	19.722	0.037	66.270	0.406
Machilus microcarpa Hemsl.	2.216	1.135	10.402	2.889	10.037	0.059	44.892	0.146
Rhus chinensis Mill.	1.893	0.783	16.367	1.933	10.814	0.035	79.426	0.311
Melia azedarach L.	2.850	1.263	27.791	2.969	12.448	0.061	96.355	NAN
Populus × canadensis Moench.	2.730	0.825	12.114	11.061	1.382	0.212	120.189	NAN
Camptotheca acuminata Decne.	1.816	1.016	21.425	7.259	13.014	0.061	59.795	0.159
*Cinnamomum bodinier*i Levl.	1.627	0.771	15.880	2.948	9.077	0.020	91.271	0.222
Catalpa ovata G. Don	3.192	2.494	15.033	3.021	11.264	0.025	53.655	0.216
Toona sinensis (A. Juss.) Roem.	3.669	1.146	21.508	3.747	14.082	0.033	24.881	0.089
Sapium sebiferum (Linn.) Roxb.	3.100	1.474	24.371	3.168	5.630	0.050	75.124	NAN
Alangium chinense (Lour.) Harms	2.553	1.343	18.508	2.695	10.388	0.019	87.566	0.171
Cladrastis platycarpa (Maxim.) Makino	3.111	1.161	41.009	3.584	10.338	0.048	49.234	0.152
Ulmus pumila L.	1.659	0.620	20.336	3.022	5.512	0.057	68.427	0.127
Ilex macrocarpa Oliv.	1.530	0.554	28.395	6.573	39.634	0.008	82.459	NAN
Ziziphus jujuba Mill. var. *spinosa* (Bunge) Hu ex H. F. Chow	3.290	1.112	3.565	1.744	7.390	0.001	141.703	NAN
Vitex canescens Kurz	1.290	2.358	7.376	9.730	18.223	0.057	48.669	NAN
Eriobotrya japonica (Thunb.) Lindl.	1.200	0.539	19.454	4.230	12.478	0.040	88.868	0.248
Morus alba L.	3.521	1.129	23.645	5.478	13.924	0.058	27.440	0.095

Prunus salicina Lindl.	1.754	0.936	25.255	3.432	18.318	0.067	53.303	0.121
Eucommia ulmoides Oliver	2.304	1.182	6.191	1.854	15.270	0.052	56.264	0.254
Platycarya strobilacea Sieb. et Zucc.	2.266	0.622	21.852	6.706	4.004	0.019	45.703	0.152
Kalopanax septemlobus (Thunb.) Koidz.	1.664	0.945	17.731	5.708	7.267	0.041	33.671	0.121
Zanthoxylum armatum DC.	1.857	1.471	22.775	4.117	13.244	0.030	131.075	0.368
Pyrus calleryana	1.862	0.835	14.544	4.445	6.919	0.047	78.514	0.159
Amygdalus persica L. var.	1.974	0.943	23.177	8.613	5.234	0.056	73.947	0.140
Euonymus meaackii Rupr.	2.120	2.585	32.244	9.182	11.999	0.096	120.189	NAN
Zanthoxylum ovalifolium Wight	1.974	0.640	22.740	3.734	8.625	0.053	89.589	0.203
Cerasus scopulorum (Koehne) Yu *et* Li	1.792	1.652	10.153	3.535	16.632	0.080	75.477	NAN
Carpinus pubescens Burk.	2.044	0.750	22.611	3.221	4.535	0.075	39.256	0.108
Lithocarpus confinis Huang	1.233	0.504	9.907	2.305	4.035	0.051	146.543	0.298
Celtis sinensis Pers.	1.505	0.582	33.395	6.018	4.746	0.028	124.857	0.267
Evodia rutaecarpa (Juss.) Benth.	1.710	1.052	20.553	5.206	3.350	0.046	130.785	NAN
Machilus cavaleriei Levl.	1.799	0.736	4.576	2.295	3.873	0.033	128.742	0.292
Diospyros kaki Thunb. var. *silvestris* Makino	2.208	0.898	18.892	5.146	19.487	0.038	86.670	0.140
Rubus inopertus (Diels) Focke	2.267	1.022	13.004	6.362	9.592	0.032	38.130	0.146
Coriaria nepalensis Wall.	1.975	1.007	10.740	6.598	4.386	0.034	128.977	0.254
Celastrus orbiculatus Thunb.	1.587	0.645	57.751	8.028	6.108	0.063	154.611	0.286
Wikstroemia scytophylla Diels	2.891	1.463	32.171	5.807	17.781	0.039	34.302	0.178
Viburnum foetidum Wall. var. *ceanothoides* (C. H. Wright) Hand.-Mazz.	2.052	1.010	11.120	3.524	15.708	0.093	29.463	0.127
Hedera nepalensis K. Koch var. *sinensis* (Tobl.) Rehd.	1.776	0.990	22.427	3.897	12.692	0.052	47.895	0.241
Debregeasia longifolia (Burm. f.) Wedd.	1.750	2.185	27.551	4.012	17.235	0.052	70.036	NAN
Rubus parvifolius L.	1.609	1.187	17.718	7.172	6.001	0.037	56.895	0.203
Rosa roxbunghii	2.710	0.851	14.005	2.576	6.710	0.035	126.989	NAN
Mallotus repandus (Willd.) Muell. Arg.	2.279	1.065	38.617	6.274	9.152	0.058	44.193	0.165
Mahonia bealei (Fort.) Carr.	1.588	1.128	3.668	1.921	12.520	0.035	107.006	0.356
Fallopia multiflora (Thunb.) Harald.	1.774	1.170	17.305	14.264	15.485	0.038	47.187	0.216

Conyza canadensis (L.) Cronq.	4.065	1.634	15.811	4.141	17.740	0.986	24.732	0.108
Ipomoea batatas (L.) Lam.	1.865	1.129	24.040	5.482	23.532	0.061	62.852	0.279
Senecio scandens Buch.-Ham. ex D. Don	2.662	2.717	16.101	3.235	18.597	0.017	53.808	0.140
Vitis piasezkii Maxim.	1.916	1.128	11.997	3.851	16.682	0.038	34.221	0.140
Clematis urophylla Franch.	1.748	1.005	18.976	6.320	16.973	0.051	146.013	0.102
Bauhinia glauca (Wall. ex Benth.) Benth.	2.288	0.816	29.374	3.691	6.523	0.075	49.821	0.127
Caesalpinia decapetala (Roth) Alston	2.545	1.000	27.642	3.815	11.993	0.053	56.817	0.184
Paederia scandens (Lour.) Merr.	2.137	0.933	14.203	6.100	16.184	0.035	40.450	0.191
Cyclosorus parasiticus (L.) Farwell.	2.316	1.120	8.950	6.256	19.523	0.066	40.863	0.152
Cyrtomium fortunei J. Sm.	2.725	0.976	4.746	4.508	21.281	0.100	33.878	0.121
Pteris vittata L.	2.261	0.830	4.202	2.218	11.766	0.035	57.897	0.140

Note that all auxiliary dataset were shared from "Functional Trait database of terrestrial ecosystems in China (China_Trait)", and the associated data presented in this table were averaged values.

Supplementary Table S3. Pearson correlations of the area-based photosynthetic capacity (A_{sat}, V_{cmax}, and J_{max}) with leaf nutrient contents and leaf thickness (*LT*). A_{sat}: the light-saturated net photosynthesis; V_{cmax}: the maximum carboxylation rate; J_{max}: the maximum electron transport rate.

	N	P	Ca	Mg	K	Na	*LT*	*LMA*
A_{sat}	-0.008	0.258*	-0.103	0.089	0.018	-0.087	0.146	-0.113
V_{cmax}	0.016	0.287*	-0.052	0.117	0.025	-0.104	0.186	-0.070
J_{max}	0.095	0.292*	0.008	0.178	0.106	-0.101	0.167	-0.029

**P<0.01 (2-tailed), *P<0.05 (2-tailed).

Supplementary Table S4. Coefficients of multivariate stepwise regression. V_{cmax}: the maximum carboxylation rate; J_{max}: the maximum electron transport rate.

	V_{cmax}			J_{max}		
Variance	AIC	*P*	R^2	AIC	*P*	R^2
N	-62.26	<0.001	0.275	-2.15	<0.001	0.299
N,Mg	-69.71	<0.001	0.395	-12.31	<0.001	0.440
N,Mg,*LT*	-73.59	<0.001	0.431	-12.22	<0.001	0.490
N,Mg,*LT*,P	-66.54	<0.001	0.491	-9.34	<0.001	0.530

Supplementary Table S5. Pearson correlations of mass-based leaf nutrient contents and leaf thickness (*LT*)

	N	P	Ca	Mg	K	Na	*LT*	*LMA*
N	1	0.476**	0.023	-0.081	0.419**	0.394**	-0.414**	-0.400**
P		1	-0.039	0.080	0.480**	0.131	0.066	-0.269*
Ca			1	0.317**	-0.121	-0.048	0.065	0.094
Mg				1	0.093	-0.033	-0.160	0.003
K					1	0.198	0.003	-0.085
Na						1	-0. 211	-0. 213
LT							1	0.594**
LMA								1

**$P<0.01$ (2-tailed), *$P<0.05$ (2-tailed).

Supplementary Table S6. Pearson correlations of mass-based photosynthetic capacity (V_{cmax} and J_{max}) with leaf nutrient contents and leaf thickness (*LT*). A_{sat}: the light-saturated net photosynthesis; V_{cmax}: the maximum carboxylation rate; J_{max}: the maximum electron transport rate.

	N	P	Ca	Mg	K	Na	*LT*	*LMA*
A_{sat}	0.525**	0.455**	-0.005	0.298*	0.310*	0.395*	-0.391**	-0.722**
V_{cmax}	0.524**	0.436**	0.014	0.303*	0.299*	0.356**	-0.401**	-0.727**
J_{max}	0.546**	0.403**	0.020	0.330**	0.336**	0.339**	-0. 442**	-0.759**

**P<0.01 (2-tailed), *P<0.05 (2-tailed).

Supplementary Table S7. Characteristics of soil and leaf in this study site. TN, Total nitrogen; TP, total phosphorus; TCa, total calcium; TMg, total magnesium; TK, total potassium; TNa, total sodium; AN, available soil nitrogen; AP, available soil phosphorus.

	TN	TP	TCa	TMg	Tk	TNa	AN	AP
Soil Content (mg g^{-1})	7.30±0.66	1.18±0.35	36.48±34.95	22.64±14.51	——	——	69.76±5.96	9.05±5.64
Soil storage (t hm^{-2})	12.04	1.68	——	——	——	——	1.2	1.26
Leaf content (mg g^{-1})	23.39±6.72	1.11±0.50	19.25±10.33	4.61±2.39	12.00±6.15	0.07±0.12	——	——

The nutrient storage of soil was reported by Chen et al. (Chen & Zhou 2017) in this study site.

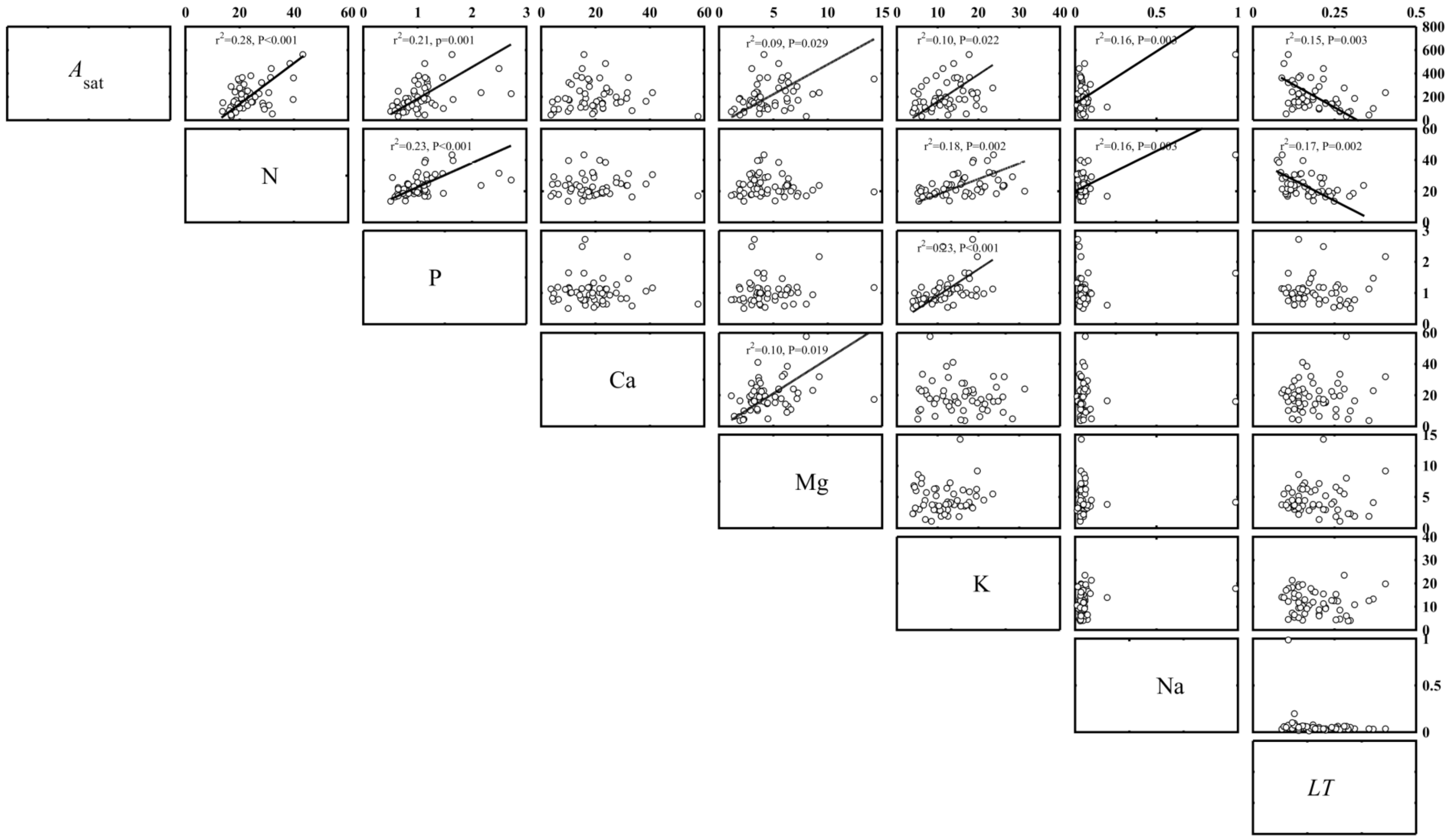

Figure S1. Relationships between of the A_{sat} (nmol g^{-1} s^{-1}) and leaf traits. A_{sat}, the light-saturated net photosynthesis; *LT*, leaf thickness (mm); Leaf nutrients (mg g^{-1}).

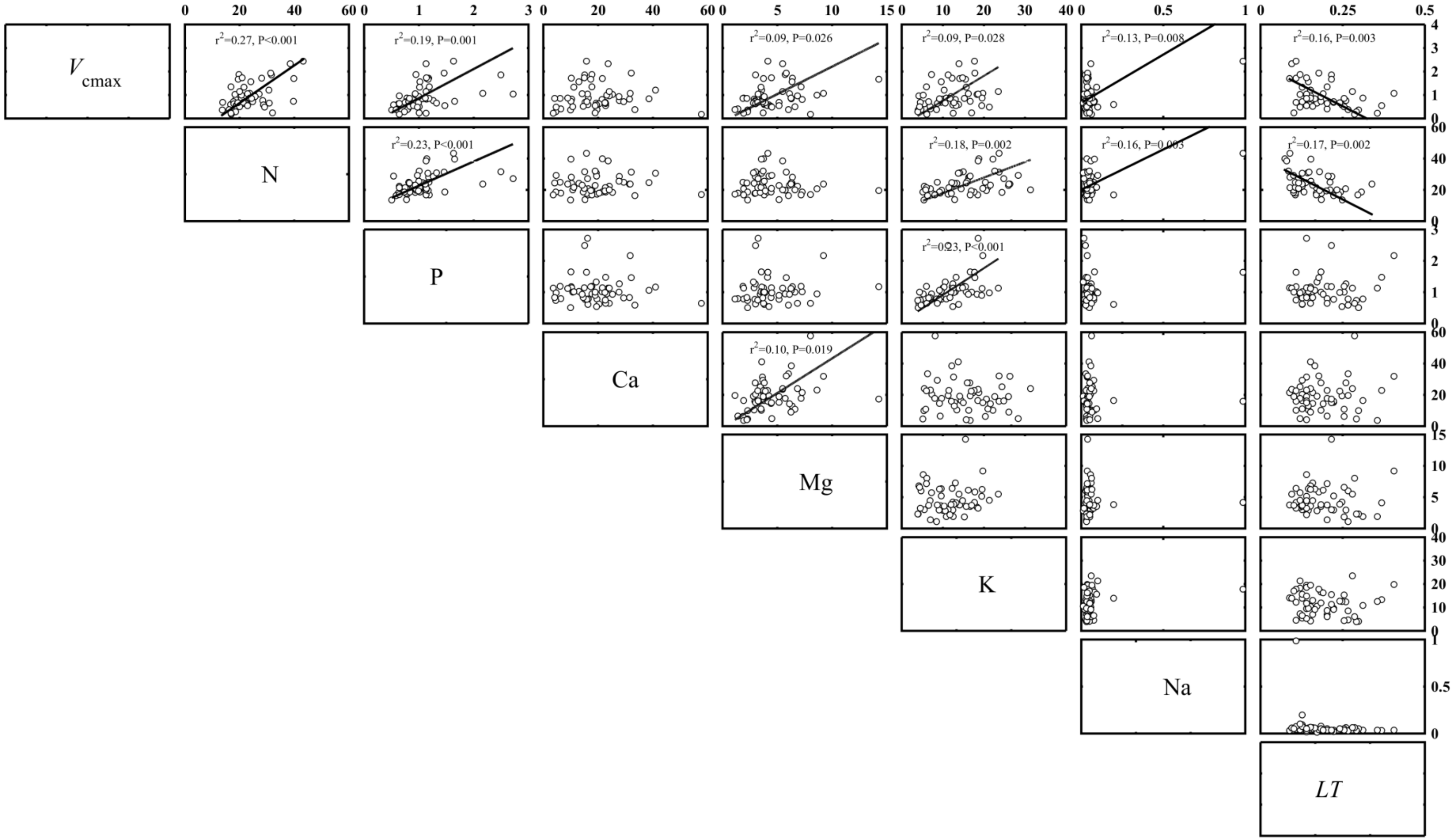

Figure S2. Relationships between of V_{cmax} (μmol g^{-1} s^{-1}) and leaf traits. V_{cmax}, the maximum carboxylation rate; *LT*, leaf thickness (mm); Leaf nutrients (mg g^{-1}).

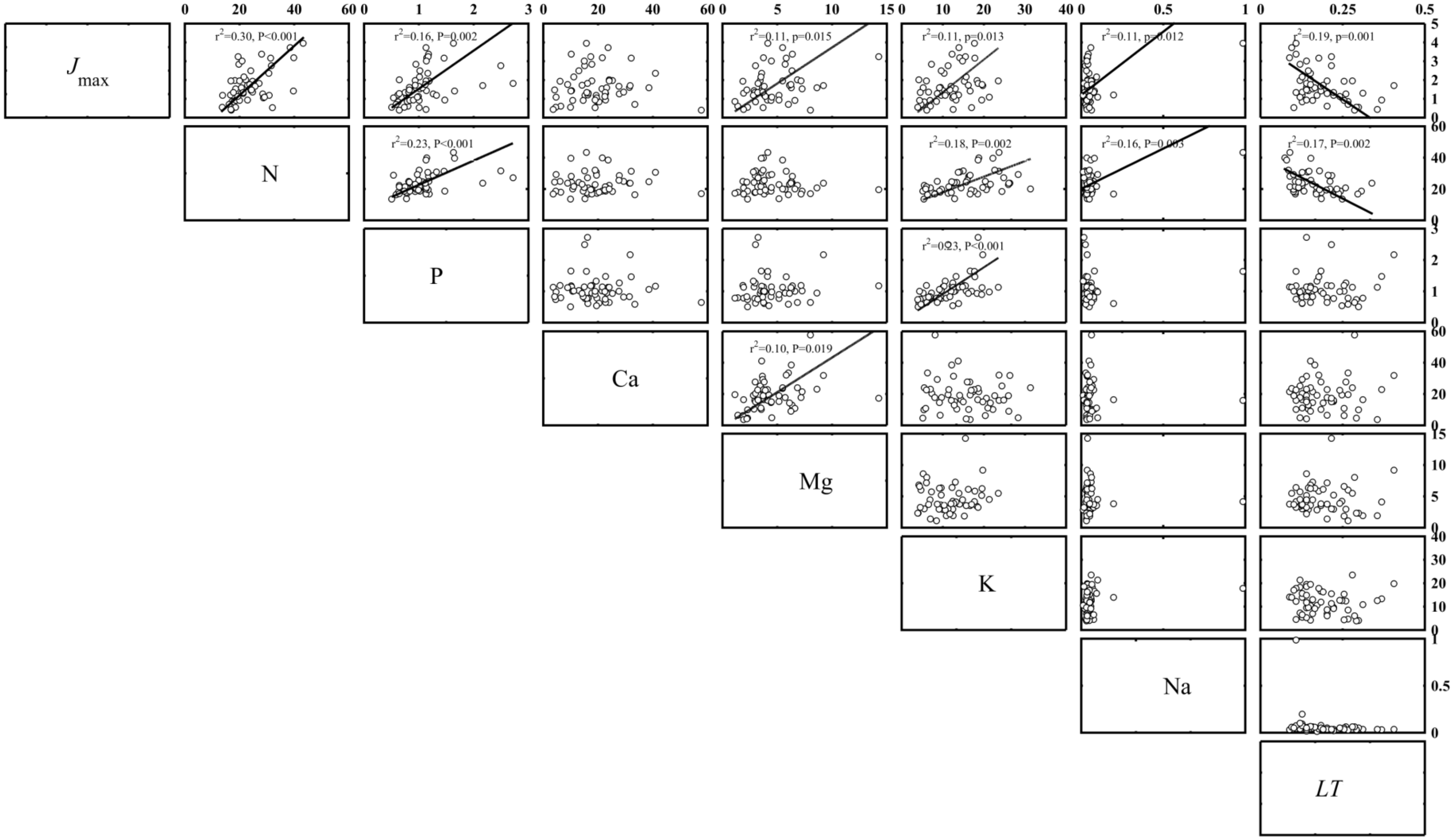

Figure S3. Relationships between of J_{max} (μmol g^{-1} s^{-1}) with leaf traits(mg g^{-1}). J_{max}, the maximum electron transport rate; *LT*, leaf thickness (mm); Leaf nutrients (mg g^{-1}).

Reference

Chen, P. & Zhou, Y. (2017). Soil nutrient capacity and forest tree sustainability in plateau Karst region. *Earth and Enviroment*, 45, 32-37 (2017). (In Chinese)
